# RETRACTION: Prevalence of Surgical Site Wound Infection After Spine Surgery in Nasal Colonization of Methicillin‐Resistant *Staphylococcus aureus* : A Meta‐Analysis

**DOI:** 10.1111/iwj.70265

**Published:** 2025-02-25

## Abstract

**RETRACTION:** ImamM. S. , Abdel‐SattarR. M. , AlqarniF. , AljumayiS. Y. S. , AltukhaisI. , AltukhaysA. S. , and AbdelrahimM. E. A. , “Prevalence of Surgical Site Wound Infection After Spine Surgery in Nasal Colonization of Methicillin‐Resistant Staphylococcus aureus: A Meta‐Analysis,” International Wound Journal 21, no. 3 (2024): e14470, 10.1111/iwj.14470.37909167 PMC10898389

The above article, published online on 01 November 2023, in Wiley Online Library (http://onlinelibrary.wiley.com/), has been retracted by agreement between the journal Editor in Chief, Professor Keith Harding; and John Wiley & Sons Ltd. Following an investigation by the publisher, all parties have concluded that this article was accepted solely on the basis of a compromised peer review process. In addition, the investigation found significant textual overlap between the methods and discussions sections of this article and another article by different authors [1]. The editors have therefore decided to retract the article. The authors did not respond to our notice regarding the retraction.

**Reference:**

[1] NingJ. , WangJ. , ZhangS. , and ShaX. “Nasal colonization of Staphylococcus aureus and the risk of surgical site infection after spine surgery: a meta‐analysis” The Spine Journal 20, no. 3 (2020): 448–456, 10.1016/j.spinee.2019.10.009.31669610

**RETRACTION:** M. S. Imam , R. M. Abdel‐Sattar , F. Alqarni , S. Y. S. Aljumayi , I. Altukhais , A. S. Altukhays , and M. E. A. Abdelrahim , “Prevalence of Surgical Site Wound Infection After Spine Surgery in Nasal Colonization of Methicillin‐Resistant Staphylococcus aureus: A Meta‐Analysis,” International Wound Journal 21, no. 3 (2024): e14470, 10.1111/iwj.14470.37909167 PMC10898389

The above article, published online on 01 November 2023, in Wiley Online Library (http://onlinelibrary.wiley.com/), has been retracted by agreement between the journal Editor in Chief, Professor Keith Harding; and John Wiley & Sons Ltd. Following an investigation by the publisher, all parties have concluded that this article was accepted solely on the basis of a compromised peer review process. In addition, the investigation found significant textual overlap between the methods and discussions sections of this article and another article by different authors [1]. The editors have therefore decided to retract the article. The authors did not respond to our notice regarding the retraction.

## Reference

[1] J. Ning , J. Wang , S. Zhang , and X. Sha “Nasal colonization of Staphylococcus aureus and the risk of surgical site infection after spine surgery: a meta‐analysis” The Spine Journal 20, no. 3 (2020): 448–456, 10.1016/j.spinee.2019.10.009.31669610

